# Thriving at Work and Associated Factors Among Nurses at a Chinese Stomatological Hospital: A Cross‐Sectional Study

**DOI:** 10.1155/jonm/7301771

**Published:** 2026-09-15

**Authors:** Qingying Zhu, Xiaping Wang, Junjie Wang, Liqing Su, Yanhong Zhang, Yi Zhang, Qianju Wu

**Affiliations:** ^1^ Department of Prosthodontics II, Xiamen Key Laboratory of Stomatological Disease Diagnosis and Treatment, Stomatological Hospital of Xiamen Medical College, No. 1309 Lvling Road Huli District, Xiamen 361008, Fujian, China

**Keywords:** empowering leadership, nursing management, oral healthcare nurses, personality traits, thriving at work

## Abstract

**Aim:**

To describe thriving at work among oral healthcare nurses and examine its associations with personality traits, empowering leadership, and department.

**Background:**

Evidence on thriving at work in oral healthcare nursing and across dental departments remains limited.

**Methods:**

A single‐center cross‐sectional census survey was conducted in a Chinese stomatological hospital. Data from 190 female nurses were analyzed using group comparisons, Pearson correlations, a Wilcoxon signed‐rank test, and standard multiple linear regression.

**Results:**

The mean total Thriving at Work Scale score was 52.42 ± 8.89, and Learning scores were higher than Vitality scores. The regression model explained 51.9% of the variance. Neuroticism was negatively associated with thriving, whereas Conscientiousness and Extraversion were positively associated with it. After simultaneous adjustment, nurses in Pediatric Dentistry had lower thriving scores than those in General Dentistry. The association with empowering leadership was less stable across sensitivity analyses.

**Conclusions:**

Thriving was associated with individual dispositions and departments in this single‐center sample; causal inferences cannot be made.

**Implications for Nursing Management:**

Managers may consider assessing both Learning and Vitality and further investigating the lower thriving observed in Pediatric Dentistry. Personality findings should be used only to guide equitable, non‐stigmatizing support.

## 1. Introduction

Thriving at work is a positive psychological state characterized by the joint experience of Vitality and Learning [1–3]. Vitality refers to feeling energized and alive at work, whereas learning reflects perceived growth through acquiring and applying knowledge and skills. Both dimensions are necessary because employees may continue to learn while feeling depleted or may feel energetic without developing professionally. The socially embedded model proposes that workplace features support thriving by enabling agentic work behaviors and access to knowledge, positive meaning, positive affect, and relational resources [1, 2]. Within this framework, personality traits may shape how employees respond to work demands and use available resources, whereas empowering leadership and department‐specific conditions represent contextual influences. In nursing populations, thriving has been associated with structural empowerment, organizational culture, work engagement, job performance, lower stress symptoms, and lower turnover intentions [4–8].

Oral health‐related nursing work varies substantially by setting. In general hospitals, nurses provide oral care to hospitalized patients [9, 10]. In oral healthcare services, dental nurses may undertake telephone triage and use clinical information systems [11], while dental outpatient work involves close patient contact and infection‐control considerations [12]. Dental assistants also provide chairside and procedure‐related support [13]. These role differences suggest that workflow and resource demands may vary across dental departments. However, dental workforce research has focused mainly on burnout, stress, depressive symptoms, and general well‐being [14, 15], and evidence on the combined experience of Learning and Vitality among nurses working in oral healthcare remains limited.

Personality traits and empowering leadership may be relevant to thriving through different pathways. Nursing studies have described personality profiles among specific nursing groups [16] and linked personality traits with perceived work environments, compassion fatigue, emotional regulation, happiness, coping, and occupational well‐being [17–20]. Conscientiousness may support persistence and self‐regulation, Extraversion may facilitate positive affect and access to relational resources, and Neuroticism may increase sensitivity to stress. Leadership represents a distinct contextual influence. Among emergency department nurses, empowering leadership was positively associated with thriving through intrinsic motivation [21]. Other nursing evidence has examined leadership in relation to interprofessional collaboration, occupational burnout, work motivation, and well‐being [22–24]. It remains unclear whether personality traits, empowering leadership, and department are independently associated with thriving among nurses working in oral healthcare.

This study therefore aimed to describe overall thriving, Learning, and Vitality among oral healthcare nurses and to examine their associations with Big Five personality traits, perceived empowering leadership, and department. The study addressed three questions: (1) what were the observed scores for overall thriving, Learning, and Vitality, and did Learning and Vitality differ within participants; (2) how were personality traits and empowering leadership associated with thriving; and (3) did departmental differences remain after adjustment for personality traits and empowering leadership?

## 2. Materials and Methods

### 2.1. Study Design and Participants

This single‐center cross‐sectional survey was conducted at the Stomatological Hospital of Xiamen Medical College. In this study, the term “oral healthcare nurses” refers to licensed nurses working in the clinical and support departments of this stomatological hospital. Data were collected from September 11 to September 12, 2025, after ethics approval had been obtained. A census approach was adopted, and all nurses employed at the hospital were screened for eligibility. Nurses were eligible if they held a valid nursing license, had at least 1 year of clinical nursing experience, and voluntarily provided electronic informed consent. Nurses who were on maternity leave or were away from their usual clinical position for advanced study during the survey period were excluded. Participant flow and questionnaire disposition are described in Section 2.4. This report was prepared in accordance with the Strengthening the Reporting of Observational Studies in Epidemiology (STROBE) recommendations for cross‐sectional studies [25].

### 2.2. Sample Size Considerations

Because a census approach was used, all eligible nurses were invited to participate, and no smaller target sample was selected in advance. Because no sufficiently comparable previous study was available to provide a reliable anticipated effect size for the same population and predictor set, a sensitivity power analysis was conducted using G∗Power Version 3.1.9.7 [26]. For a fixed‐effects multiple regression model with 14 predictors, a total sample size of 190, *α* = 0.05, and 80% power, the minimum detectable overall regression effect was Cohen’s *f* ^2^ = 0.103, equivalent to *R* ^2^ = 0.093. Thus, the available sample was sufficient to detect an overall effect explaining approximately 9.3% or more of the variance, whereas smaller effects may not have been detected.

### 2.3. Instruments

#### 2.3.1. General Information Questionnaire

A self‐designed questionnaire was developed based on the study objectives to collect nurses’ demographic and occupational characteristics, including sex, age, educational level, marital status, number of children, professional title, years of service in the hospital, average daily working hours, department, and employment status.

#### 2.3.2. Thriving at Work Scale (TWS)

The TWS was originally developed by Porath et al. [3] and subsequently translated, culturally adapted, and psychometrically evaluated among Chinese employees by Zeng et al. [27]. The Chinese version comprises 10 items across two dimensions, Learning and Vitality, with five items in each dimension. Items are rated on a seven‐point Likert scale ranging from 1 (completely inconsistent) to 7 (completely consistent), with Items 4 and 8 reverse‐scored. Item scores are summed to produce a raw total score ranging from 10 to 70, whereas each dimension score ranges from 5 to 35; higher scores indicate greater thriving at work. In the Chinese validation study, Cronbach’s *α* was 0.88 for the total scale, 0.82 for the Learning subscale, and 0.81 for the Vitality subscale. For descriptive comparability, mean item scores were additionally reported in Table 1. The raw total TWS score was designated as the primary outcome for the multivariable analysis because thriving is conceptualized as the joint experience of Learning and Vitality. In the present study, Cronbach’s *α* was 0.873 for the total TWS, 0.706 for the Learning subscale, and 0.819 for the Vitality subscale, indicating acceptable to good internal consistency.

**TABLE 1:** Descriptive statistics for study variables.

Scales and subdimensions	Number of items	Min	Max	Total score (mean ± SD)	Mean item score (mean ± SD)
Empowering leadership total score	12	16	60	43.65 ± 8.20	3.64 ± 0.68
Work Meaningfulness	3	3	15	11.14 ± 2.46	3.71 ± 0.82
Participation in Decision‐Making	3	4	15	10.91 ± 2.38	3.64 ± 0.79
Confidence in Employees’ Competence	3	3	15	11.00 ± 2.18	3.67 ± 0.73
Provision of Support	3	5	15	10.69 ± 2.13	3.56 ± 0.71
CBF‐PI‐B					
Neuroticism	8	8	47	21.43 ± 7.26	2.68 ± 0.91
Conscientiousness	8	13	48	36.13 ± 6.27	4.52 ± 0.78
Agreeableness	8	22	48	36.21 ± 6.06	4.53 ± 0.76
Openness	8	8	48	30.35 ± 7.14	3.79 ± 0.89
Extraversion	8	9	47	27.62 ± 6.80	3.45 ± 0.85
TWS	10	28	70	52.42 ± 8.89	5.24 ± 0.89
Learning	5	19	35	27.28 ± 4.40	5.46 ± 0.88
Vitality	5	9	35	25.14 ± 5.41	5.03 ± 1.08

*Note:* CBF‐PI‐B, Chinese Big Five Personality Inventory–Brief Version; minimum and maximum values represent the observed values in the study sample.

Abbreviations: SD, standard deviation; TWS, Thriving at Work Scale.

#### 2.3.3. Chinese Big Five Personality Inventory–Brief Version (CBF‐PI‐B)

The CBF‐PI‐B was developed by Wang, Dai, and Yao within the Chinese Big Five personality framework and is conceptually related to the five‐factor model represented by the NEO‐PI‐R [28, 29]. The instrument comprises 40 items assessing five personality dimensions: Neuroticism, Conscientiousness, Agreeableness, Openness, and Extraversion. Each dimension contains eight items rated on a six‐point Likert scale, producing a subscale score ranging from 8 to 48. Higher subscale scores indicate a higher level of the corresponding personality trait. Because the five dimensions represent conceptually distinct personality traits, an overall composite personality score was not calculated or used in the analyses. Previous studies have demonstrated satisfactory reliability and validity of the CBF‐PI‐B. In the present study, Cronbach’s *α* coefficients for the five subscales ranged from 0.835 to 0.920, indicating good internal consistency.

#### 2.3.4. Leadership Empowerment Behavior (LEB) Measure

Empowering leadership was assessed using the 12‐item LEB measure developed by Ahearne et al. [30] and subsequently adapted by Song for use among Chinese hospital nurses [31]. Song adapted the measure to the nursing context through translation and back‐translation and expert review. The adapted measure comprises four three‐item dimensions: Work Meaningfulness, Participation in Decision‐Making, Confidence in Employees’ Competence, and Provision of Support. Items are rated on a five‐point Likert scale ranging from 1 (completely uncharacteristic) to 5 (highly characteristic). Item scores are summed to yield a total score ranging from 12 to 60, with higher scores indicating a higher perceived level of empowering leadership. In Song’s formal survey of 393 nurses, Cronbach’s *α* was 0.922 for the total measure, with coefficients of 0.875–0.915 across the four dimensions. In the present study, Cronbach’s *α* for the total measure was 0.942.

### 2.4. Data Collection Procedure

The electronic questionnaire was distributed by trained investigators using a standardized guidance script describing the study purpose, procedures, voluntary nature of participation, and completion instructions. Participants were informed that there were no right or wrong answers and were asked to respond according to their actual workplace experiences. Responses were collected confidentially and analyzed in de‐identified form. To limit duplicate submissions, the survey platform permitted only one submission from each device. All questionnaire items were mandatory, and the estimated completion time was 10–12 min. These procedural measures were used to reduce evaluation apprehension, socially desirable responding, and duplicate responses.

At the time of the survey, 238 nurses were employed at the hospital. After 16 nurses with less than 1 year of clinical nursing experience, three nurses on maternity leave, and two nurses engaged in advanced study were excluded, 217 nurses were eligible and invited to participate. A total of 193 nurses returned the questionnaire, corresponding to a response rate of 88.9%. Before scale scores were calculated, responses were screened for invariant responding across the 62 Likert‐scale items. Three questionnaires in which the same response category was selected for every item were flagged as probable straight‐lining and excluded using a uniformly applied data‐quality rule. No other questionnaires met the exclusion criterion. Consequently, 190 valid questionnaires were included, representing 98.4% of all returned questionnaires. All participants included in the final analytic sample were female. Because all electronic questionnaire items were mandatory, the final dataset contained no item‐level missing values.

### 2.5. Data Analysis

Questionnaire data were independently checked by two researchers, reverse‐scored according to the validated scoring instructions, and analyzed using IBM SPSS Statistics version 26.0. Continuous variables are reported as means and standard deviations and categorical variables as frequencies and percentages. Distributions were assessed using histograms, skewness, and kurtosis; absolute skewness below 2 and absolute kurtosis below 7 indicated approximate normality [32, 33].

Learning and Vitality were compared using the Wilcoxon signed‐rank test because their paired differences were not normally distributed. Effect size was calculated as *r* = |*Z*|/√*N*, where N was the number of non‐zero paired differences. Group differences in total TWS scores were examined using independent‐samples *t*‐tests or one‐way analysis of variance. When variances were unequal, Welch’s analysis of variance and Tamhane’s T2 pairwise tests were used. Cohen’s *d* and *η* ^2^ were reported as effect sizes.

Pearson correlations were calculated among total TWS, Learning, Vitality, the five personality traits, and empowering leadership. As a supporting assessment of potential common method variance, all 62 items were entered into an unrotated principal component analysis. Because Harman’s single‐factor procedure cannot establish the absence of common method bias, the result was interpreted only as a limited diagnostic check [34].

A standard multiple linear regression used total TWS as the dependent variable. The five personality traits, empowering leadership, and eight department dummy variables were entered simultaneously, with General Dentistry as the reference group. Sex was omitted because all participants were female. The raw total TWS score was used as the outcome in the primary multivariable model because thriving is theoretically defined by the joint experience of Learning and Vitality. The two dimensions were examined separately in descriptive, within‐participant, and correlation analyses to characterize their observed patterns. Separate adjusted models for Learning and Vitality were not part of the primary analytic plan and were not added post hoc because they would constitute additional exploratory models, increase analytic multiplicity, and require cautious interpretation given the small sample sizes in several departments. Results are reported as *B*, standard error, *β*, 95% confidence interval, and *p* value. Regression diagnostics included tolerance and variance inflation factors to assess multicollinearity, residual plots to examine linearity and homoscedasticity, standardized residuals to identify potential outlying observations, and Cook’s distance to assess influential observations. Sensitivity analyses excluded, separately, the four Nursing Department participants and the two observations with absolute standardized residuals greater than 3.0. An additional sensitivity analysis further adjusted the primary model for years of service and daily working hours, selected as theoretically relevant occupational covariates irrespective of their univariable statistical significance. Years of service and daily working hours were entered as categorical dummy variables, with 1–5 years and 7–8 h as the respective reference categories. All tests were two‐sided, with *p* < 0.05.

### 2.6. Ethical Considerations

This study was conducted in strict accordance with the ethical principles of the Declaration of Helsinki. The study protocol was reviewed and approved by the Ethics Committee of the Stomatological Hospital of Xiamen Medical College (Approval No.: KS20250909001). All participants provided electronic informed consent by selecting the option “I agree to participate in this study” before accessing the questionnaire. Research data were used solely for scientific purposes, stored securely, and analyzed in de‐identified form.

## 3. Results

### 3.1. General Characteristics and Thriving at Work

All 190 participants included in the final analysis were female. Table 2 presents the distributions of demographic and occupational characteristics and their associations with raw total TWS scores. Age, educational level, marital status, number of children, professional title, years of service in the hospital, daily working hours, and employment status were not significantly associated with TWS scores (all *p* > 0.05). Department of work was the only characteristic showing a statistically significant overall association with thriving at work. Because the homogeneity‐of‐variance assumption was violated, Welch’s analysis of variance was used and indicated a significant difference in raw total TWS scores across departments, Welch’s *F* (8, 38.974) = 4.102, *p* = 0.001, *η* ^2^ = 0.110. The unadjusted department comparison yielded an effect size of *η* ^2^ = 0.110; however, this estimate should be interpreted cautiously because several departmental groups were small and group variances were unequal. Tamhane’s T2 pairwise comparisons showed that nurses working in General Dentistry had higher raw total TWS scores than those working in Pediatric Dentistry, with a mean difference of 8.668 points (95% *CI*, 2.35–14.98; *p* = 0.001). Nurses working in the Central Sterile Supply Department also had higher raw total TWS scores than those working in Pediatric Dentistry, with a mean difference of 11.907 points (95% *CI*, 3.49–20.32; *p* = 0.001). No other pairwise comparisons were statistically significant. These department‐specific findings should be interpreted cautiously because several departments had relatively small sample sizes.

**TABLE 2:** Thriving at work scale scores by demographic and occupational characteristics of the participants.

Variables	Category	*N*	%	TWS scores (mean ± SD)	*p*	Effect size
Age					0.950^b^	0.002
	20–29 years	78	41.1	52.87 ± 9.00		
	30–39 years	86	45.3	52.13 ± 8.88		
	40–49 years	20	10.5	51.95 ± 8.03		
	≥ 50 years	6	3.2	52.17 ± 12.16		
Education level					0.491^a^	0.13
	Junior college	32	16.8	53.41 ± 8.48		
	Bachelor’s degree and above	158	83.2	52.22 ± 8.98		
Marital status					0.961^a^	0.01
	Single	70	36.8	52.46 ± 8.95		
	Married	120	63.2	52.39 ± 8.89		
Number of children					0.911^b^	0.001
	None	83	43.7	52.17 ± 9.06		
	One	62	32.6	52.81 ± 8.94		
	Two or more	45	23.7	52.33 ± 8.68		
Professional title					0.671^b^	0.008
	Registered Nurse	37	19.5	51.38 ± 9.29		
	Senior Nurse	90	47.4	52.71 ± 8.21		
	Supervisor Nurse	51	26.8	52.08 ± 10.04		
	Associate Chief Nurse or higher	12	6.3	54.83 ± 7.73		
Years of service in the hospital					0.670^b^	0.004
	1–5 years	71	37.4	51.86 ± 8.85		
	6–10 years	63	33.2	52.29 ± 8.92		
	≥ 11 years	56	29.5	53.27 ± 8.99		
Department of Work					0.001**^c^**	0.110
	General Dentistry	45	23.7	54.84 ± 7.94		
	Pediatric Dentistry	17	8.9	46.18 ± 5.81		
	Prosthodontics	17	8.9	51.29 ± 7.59		
	Orthodontics	15	7.9	49.33 ± 10.34		
	Endodontics	38	20.0	53.61 ± 10.55		
	Oral and Maxillofacial Surgery	27	14.2	50.78 ± 7.78		
	Oral and Maxillofacial Surgery Ward	15	7.9	52.80 ± 7.12		
	Central Sterile Supply Department	12	6.3	58.08 ± 6.36		
	Nursing Department	4	2.1	49.25 ± 15.35		
Daily working hours					0.680^b^	0.004
	< 7 h	8	4.2	55.13 ± 9.36		
	7–8 h	144	75.8	52.28 ± 8.62		
	> 8 h	38	20.0	52.37 ± 9.90		
Employment status					0.694^b^	0.004
	Non‐staff contract employment	116	61.1	51.97 ± 8.91		
	Labor‐dispatch employment	66	34.7	53.09 ± 8.75		
	Public institution staffing	8	4.2	53.25 ± 10.47		

*Note:* Statistically significant *p* values are presented in bold. Effect sizes are reported as Cohen’s *d* for two‐group comparisons and eta squared (*η* ^2^) for comparisons involving three or more groups. For the department comparison, *η* ^2^ was calculated descriptively from the conventional between‐group and total sums of squares, whereas statistical significance was evaluated using Welch’s analysis of variance because group variances were unequal. The bold value (*p* = 0.001) indicates a statistically significant difference in TWS scores among departments of work (*p* < 0.05).

Abbreviations: SD, standard deviation; TWS, Thriving at Work Scale.

^a^Independent‐samples *t*‐test.

^b^Conventional one‐way analysis of variance.

^c^Welch’s analysis of variance because the homogeneity‐of‐variance assumption was violated; significant pairwise differences were examined using Tamhane’s T2 test.

### 3.2. Descriptive Statistics of Empowering Leadership, Personality Traits, and Thriving at Work

Descriptive statistics for the empowering leadership, CBF‐PI‐B, and TWS are presented in Table 1. The mean item score for empowering leadership was 3.64 ± 0.68. Among the four empowering leadership dimensions, Work Meaningfulness had the highest mean item score (3.71 ± 0.82), followed by Confidence in Employees’ Competence (3.67 ± 0.73) and Participation in Decision‐Making (3.64 ± 0.79). Provision of Support had the lowest mean item score (3.56 ± 0.71).

Among the personality dimensions, Agreeableness (4.53 ± 0.76) and Conscientiousness (4.52 ± 0.78) had the highest mean item scores, followed by Openness (3.79 ± 0.89), Extraversion (3.45 ± 0.85), and Neuroticism (2.68 ± 0.91).

The mean raw total TWS score was 52.42 ± 8.89, corresponding to a mean item score of 5.24 ± 0.89. The mean raw dimension scores were 27.28 ± 4.40 for Learning and 25.14 ± 5.41 for Vitality, corresponding to mean item scores of 5.46 ± 0.88 and 5.03 ± 1.08, respectively. Because the paired differences were not normally distributed, the Learning and Vitality scores were compared using the Wilcoxon signed‐rank test. Learning scores were significantly higher than Vitality scores (*Z* = −6.275, *p* < 0.001, *r* = 0.51, calculated using the number of non‐zero paired differences).

In the supporting Harman assessment, the first unrotated component accounted for 28.227% of the total variance. This finding did not indicate that a single component dominated the item covariance structure but did not rule out common method bias.

### 3.3. Correlation Analysis

Pearson correlation coefficients are presented in Table 3. The raw total TWS score was positively correlated with Conscientiousness (*r* = 0.581, *p* < 0.01), Agreeableness (*r* = 0.507, *p* < 0.01), Openness (*r* = 0.391, *p* < 0.01), Extraversion (*r* = 0.370, *p* < 0.01), and the empowering leadership total score (*r* = 0.449, *p* < 0.01), and was negatively correlated with Neuroticism (*r* = −0.487, *p* < 0.01). Learning and Vitality showed similar correlation patterns: both dimensions were positively correlated with Conscientiousness, Agreeableness, Openness, Extraversion, and empowering leadership and negatively correlated with Neuroticism.

**TABLE 3:** Correlations among thriving, personality traits, and empowering leadership.

Variables	1	2	3	4	5	6	7	8	9
1	1								
2	0.884^∗∗^	1							
3	0.925^∗∗^	0.640^∗∗^	1						
4	−0.487^∗∗^	−0.409^∗∗^	−0.469^∗∗^	1					
5	0.581^∗∗^	0.466^∗∗^	0.576^∗∗^	−0.426^∗∗^	1				
6	0.507^∗∗^	0.399^∗∗^	0.509^∗∗^	−0.372^∗∗^	0.681^∗∗^	1			
7	0.391^∗∗^	0.297^∗∗^	0.401^∗∗^	−0.280^∗∗^	0.490^∗∗^	0.411^∗∗^	1		
8	0.370^∗∗^	0.272^∗∗^	0.388^∗∗^	−0.180^∗^	0.316^∗∗^	0.324^∗∗^	0.479^∗∗^	1	
9	0.449^∗∗^	0.311^∗∗^	0.486^∗∗^	−0.258^∗∗^	0.457^∗∗^	0.442^∗∗^	0.310^∗∗^	0.234^∗∗^	1

*Note: N* = 190; 1 = Thriving at Work Scale; 2 = Learning; 3 = Vitality; 4 = Neuroticism; 5 = Conscientiousness; 6 = Agreeableness; 7 = Openness; 8 = Extraversion; 9 = Empowering leadership total score.

^∗^ *p* < 0.05.

^∗∗^ *p* < 0.01.

The empowering leadership total score was negatively correlated with Neuroticism (*r* = −0.258, *p* < 0.01) and positively correlated with Conscientiousness (*r* = 0.457, *p* < 0.01), Agreeableness (*r* = 0.442, *p* < 0.01), Openness (*r* = 0.310, *p* < 0.01), and Extraversion (*r* = 0.234, *p* < 0.01). Correlations among the personality dimensions ranged from weak to strong. As expected, the TWS total score was strongly correlated with Learning and Vitality because these dimensions are components of the total scale. These part–whole correlations were reported descriptively, and the TWS total score and its component dimensions were not entered simultaneously into the regression model.

### 3.4. Multiple Regression Analysis

A standard multiple linear regression was performed using the raw total TWS score as the dependent variable. The model was statistically significant, *F* (14, 175) = 13.506, *p* < 0.001, explaining 51.9% of the variance in TWS scores (adjusted *R* ^2^ = 0.481).

Neuroticism was negatively associated with thriving (*B* = −0.323; 95% *CI*, −0.468 to −0.179; *p* < 0.001), whereas Conscientiousness (*B* = 0.351; 95% *CI*, 0.124–0.578; *p* = 0.003), Extraversion (*B* = 0.192; 95% *CI*, 0.031–0.353; *p* = 0.020), and empowering leadership (*B* = 0.146; 95% *CI*, 0.013–0.280; *p* = 0.032) were positively associated with thriving. Compared with General Dentistry, Pediatric Dentistry had lower TWS scores (*B* = −4.995; 95% *CI*, −8.693 to −1.296; *p* = 0.008). Other department coefficients were not statistically significant (Table 4). Thus, after adjustment for personality traits and empowering leadership, only the difference between Pediatric Dentistry and General Dentistry remained statistically significant.

**TABLE 4:** Multiple linear regression analysis of variables associated with raw total TWS scores.

Variables	*B*	SE	*β*	*t*	*p*	95% *CI* for *B*		VIF
						Lower	Upper	
Constant	28.664	4.809		5.960	**< 0.001**	19.173	38.156	
Neuroticism	−0.323	0.073	−0.264	−4.412	**< 0.001**	−0.468	−0.179	1.306
Conscientiousness	0.351	0.115	0.248	3.052	**0.003**	0.124	0.578	2.400
Agreeableness	0.194	0.111	0.132	1.753	0.081	−0.024	0.413	2.077
Openness	−0.005	0.085	−0.004	−0.055	0.956	−0.172	0.163	1.688
Extraversion	0.192	0.082	0.147	2.357	**0.020**	0.031	0.353	1.418
Empowering leadership total score	0.146	0.068	0.135	2.161	**0.032**	0.013	0.280	1.424

*Department of work (reference: General Dentistry)*								
Pediatric Dentistry	−4.995	1.874	−0.161	−2.665	**0.008**	−8.693	−1.296	1.325
Prosthodontics	0.336	1.881	0.011	0.179	0.858	−3.377	4.049	1.336
Orthodontics	−2.258	1.973	−0.069	−1.145	0.254	−6.152	1.635	1.311
Endodontics	0.744	1.446	0.034	0.514	0.608	−2.110	3.597	1.550
Oral and maxillofacial surgery	−1.797	1.631	−0.071	−1.102	0.272	−5.015	1.421	1.502
Oral and maxillofacial surgery ward	0.539	1.937	0.016	0.278	0.781	−3.284	4.362	1.264
Central sterile supply department	2.616	2.092	0.072	1.251	0.213	−1.513	6.746	1.200
Nursing department	−4.919	3.381	−0.080	−1.455	0.147	−11.592	1.754	1.092

*Note: N* = 190. The raw total TWS score was used as the dependent variable. All predictors were entered simultaneously. The Department of work was represented by dummy variables, with General Dentistry as the reference category. Sex was not included because all participants in the final analytic sample were female. Model summary: *R* ^2^ = 0.519; adjusted *R* ^2^ = 0.481; *F* (14, 175) = 13.506; *p* < 0.001. B, unstandardized regression coefficient; *β*, standardized regression coefficient. The bold values indicate statistically significant associations, with *p* values < 0.05.

Abbreviations: CI, confidence interval; SE, standard error; TWS, Thriving at Work Scale; VIF, variance inflation factor.

Regression diagnostics did not indicate major violations of the assessed assumptions. Variance inflation factors ranged from 1.092 to 2.400, residual plots showed no clear systematic pattern, and the maximum Cook’s distance was 0.096. Two valid observations had absolute standardized residuals slightly greater than 3.0 and were retained in the primary analysis.

Sensitivity analyses excluding these two observations or the four Nursing Department participants yielded consistent findings for Neuroticism, Conscientiousness, Extraversion, and Pediatric Dentistry. The estimates for Agreeableness and empowering leadership were less stable across analyses (Supporting Tables 1 and 2). In an additional sensitivity analysis, the primary model was further adjusted for years of service and daily working hours. The main associations remained materially unchanged: Neuroticism remained negatively associated with TWS scores, whereas Conscientiousness, Extraversion, and empowering leadership remained positively associated, and Pediatric Dentistry remained associated with lower TWS scores than General Dentistry. Neither years of service nor daily working hours showed statistically significant independent associations with TWS scores. The additional covariates explained little additional variance (Δ*R* ^2^ = 0.003, *p* = 0.899; Supporting Table 3).

## 4. Discussion

This study provides preliminary evidence on thriving at work among oral healthcare nurses in a Chinese stomatological hospital. The mean total TWS score was 52.42 ± 8.89 and was interpreted descriptively because validated cut‐off values are unavailable. Learning scores were higher than Vitality scores. In the primary adjusted model, Neuroticism, Conscientiousness, Extraversion, empowering leadership, and Pediatric Dentistry were associated with thriving. Across the sensitivity analyses, Neuroticism, Conscientiousness, Extraversion, and Pediatric Dentistry were the most consistent findings, whereas the association with empowering leadership was less stable. Agreeableness was not statistically significant in the primary model and was not consistently associated across sensitivity analyses.

The higher Learning than Vitality score indicates that perceived professional growth and day‐to‐day energy were not equally strong in this sample. Dental nursing work can involve varied clinical procedures, close patient contact, triage, use of clinical information systems, and infection‐control requirements [11–13]. These features may create opportunities to acquire knowledge and skills while simultaneously imposing attentional and interpersonal demands. This interpretation is also compatible with job demands‐resources theory, which proposes that job demands consume energy, whereas job resources support motivation and well‐being [35]. Because workload, fatigue, recovery, and emotional exhaustion were not measured, these mechanisms remain hypotheses rather than demonstrated explanations. Because dimension‐specific adjusted models were not conducted, the present study cannot determine whether the independent associations of personality, leadership, and department differed between Learning and Vitality.

Nurses in Pediatric Dentistry had lower thriving scores than those in General Dentistry, and this association remained in the two exclusion‐based sensitivity analyses and in the additional covariate‐adjusted model. Pediatric dental care commonly requires age‐appropriate communication and nonpharmacological behavior‐guidance strategies [36]. These requirements may contribute to distinctive interpersonal or workflow demands, but such mechanisms were not measured in this study. The finding should therefore be interpreted cautiously because only 17 participants worked in Pediatric Dentistry and all participants came from one hospital. It identifies a department requiring further assessment rather than demonstrating that pediatric dental nursing generally results in lower thriving.

Neuroticism was negatively associated with thriving, whereas Conscientiousness and Extraversion were positively associated with it. These results are consistent with evidence linking personality traits to job satisfaction, work engagement, and occupational well‐being [37–39]. Greater stress sensitivity may make thriving more difficult to sustain, whereas persistence, self‐regulation, positive affect, and interpersonal engagement may facilitate the personal and relational resources relevant to thriving. These pathways were not measured directly, and personality should not be treated as a deterministic cause. Agreeableness and Openness were correlated with thriving but did not show stable independent associations after simultaneous adjustment, possibly because of shared variance with other traits or contextual factors.

Empowering leadership was positively associated with thriving in the primary model, but the coefficient was no longer statistically significant after the Nursing Department subgroup was excluded. This finding should therefore be considered preliminary. The measured leadership behaviors—enhancing work meaningfulness, involving nurses in decision‐making, expressing confidence in their competence, and providing support—represent potentially relevant workplace resources [21, 30, 31]. However, longitudinal or intervention studies are needed to determine whether changes in leadership precede changes in thriving. The model *R* ^2^ reflects the proportion of variance explained within this sample and should not be interpreted as evidence of causality or out‐of‐sample predictive performance.

For nursing management, the findings support attention to both components of thriving rather than reliance on the total score alone. Managers may consider assessing both nurses’ perceived learning at work and their day‐to‐day Vitality when evaluating thriving. The lower thriving observed in Pediatric Dentistry suggests that this department may warrant closer local assessment; however, the present study does not identify which departmental conditions account for this difference. Empowering leadership may also warrant further evaluation as a potentially modifiable management factor, although its association was less stable across sensitivity analyses and the cross‐sectional design does not establish the effectiveness of leadership interventions. Personality findings should be interpreted only as indicators of possible differences in support needs and should not be used for recruitment, promotion, performance appraisal, or departmental allocation. Future studies and local assessments could examine whether workload, staffing and resource adequacy, the clinical work environment, recovery opportunities, and organizational, managerial, and coworker support help explain differences in thriving across departments [4, 24, 40–43].

## 5. Limitations

This study has several limitations. First, its single‐center cross‐sectional design prevents causal or temporal inference and limits generalizability. Although a census approach achieved an 88.9% response rate, non‐response bias remains possible, and all analyzed participants were female. Second, departmental sample sizes were unequal, particularly for Pediatric Dentistry (*n* = 17) and the Nursing Department (*n* = 4), making some coefficients imprecise and sensitive to sample composition. Third, all principal variables were self‐reported at one time point. Harman’s single‐factor result did not indicate dominance by one component, but this test cannot exclude common method bias. Fourth, unmeasured factors—including workload, staffing, chairside working time, emotional labor, behavioral management demands, recovery, autonomy, team climate, burnout, and organizational support—may have produced residual confounding and limited interpretation of the Learning–Vitality difference and departmental findings. Finally, qualitative data and workplace observations were not collected. Multicenter longitudinal and mixed‐methods studies with larger, more balanced departmental samples and multiple data sources are needed.

## 6. Conclusion

Thriving among oral healthcare nurses was associated with individual dispositions and workplace context in this single‐center sample. Learning exceeded Vitality, and the most consistent adjusted associations involved Neuroticism, Conscientiousness, Extraversion, and Pediatric Dentistry. Empowering leadership showed a less stable association. Future multicenter longitudinal studies should examine whether department‐specific demands, staffing, recovery, emotional demands, and leadership contribute to differences in thriving, while ensuring that personality findings inform equitable support rather than personnel selection or evaluation.

## Author Contributions

Qingying Zhu: conceptualization, data curation, formal analysis, and writing–original draft. Xiaping Wang: conceptualization, data curation, formal analysis, and writing–original draft. Junjie Wang: methodology and writing–review and editing. Liqing Su and Yanhong Zhang: investigation and data curation. Yi Zhang: conceptualization, methodology, project administration, and writing–review & editing. Qianju Wu: supervision and writing–review & editing.

## Funding

This research was supported by the Medical and Health Guidance Project of Xiamen City and the Science and Technology Bureau of Fujian Province (Grant No. 3502Z20244ZD1336) and by the Education and Teaching Reform Projects of Xiamen Medical College (Grant Nos. XWSJ2025011 and XWSJ2025012).

## Disclosure

All authors reviewed and approved the final manuscript.

## Ethics Statement

The study protocol was reviewed and approved by the Ethics Committee of the Stomatological Hospital of Xiamen Medical College (approval no. KS20250909001) and was conducted in accordance with the Declaration of Helsinki. All participants provided electronic informed consent before accessing the questionnaire.

## Consent

Please see the Ethics Statement.

## Conflicts of Interest

The authors declare no conflicts of interest.

## Supporting Information

Additional supporting information can be found online in the Supporting Information section at the end of this article.

## Supporting information


**Supporting Information 1** Supporting Table 1. Sensitivity analysis of the multiple linear regression model after excluding two observations with absolute standardized residuals greater than 3.0.

**Supporting Information 2** Supporting Table 2. Sensitivity analysis of the multiple linear regression model after excluding the Nursing Department subgroup because of its very small sample size (*n* = 4).

**Supporting Information 3** Supporting Table 3. Sensitivity analysis of the multiple linear regression model additionally adjusting for years of service and daily working hours.

## Data Availability

The data that support the findings of this study are available from the corresponding author upon reasonable request.
