# Supplementary material for: Thriving at Work and Associated Factors Among Nurses at a Chinese Stomatological Hospital: A Cross‐Sectional Study
Source: J Nurs Manag. 2026 Sep 15;2026:7301771. doi: 10.1155/jonm/7301771 (PMC13578935; doi:10.1155/jonm/7301771)
Supplement: Supplementary file 1 [file JONM-2026-7301771-s001.docx]

**Supplementary Table 1.** Sensitivity analysis of the multiple linear regression model after excluding two observations with absolute standardized residuals greater than 3.0

**Model fit and diagnostics:** *N* = 188; R = 0.752; R² = 0.566; adjusted R² = 0.531; *F* (14, 173) = 16.113, *P* < 0.001; maximum Cook’s distance = 0.076.

| **Variables** | ***B*** | **SE** | ***β*** | ***t*** | ***P*** | **95% *CI*** | **Tolerance** | **VIF** |
| --- | --- | --- | --- | --- | --- | --- | --- | --- |
| Constant | 30.287 | 4.579 |  | 6.614 | < 0.001 | 21.249 to 39.326 |  |  |
| Neuroticism | −0.409 | 0.072 | −0.327 | −5.723 | < 0.001 | −0.551 to −0.268 | 0.771 | 1.297 |
| Conscientiousness | 0.341 | 0.110 | 0.240 | 3.101 | 0.002 | 0.124 to 0.558 | 0.419 | 2.384 |
| Agreeableness | 0.229 | 0.105 | 0.156 | 2.178 | 0.031 | 0.021 to 0.436 | 0.492 | 2.032 |
| Openness | −0.029 | 0.080 | −0.024 | −0.363 | 0.717 | −0.188 to 0.130 | 0.587 | 1.703 |
| Extraversion | 0.165 | 0.078 | 0.127 | 2.127 | 0.035 | 0.012 to 0.318 | 0.703 | 1.422 |
| Empowering leadership total score | 0.153 | 0.064 | 0.142 | 2.383 | 0.018 | 0.026 to 0.280 | 0.705 | 1.419 |
| Pediatric Dentistry | −4.510 | 1.777 | −0.147 | −2.538 | 0.012 | −8.018 to −1.002 | 0.749 | 1.335 |
| Prosthodontics | 1.014 | 1.789 | 0.033 | 0.567 | 0.572 | −2.518 to 4.545 | 0.739 | 1.353 |
| Orthodontics | −1.642 | 1.871 | −0.050 | −0.877 | 0.381 | −5.334 to 2.051 | 0.758 | 1.320 |
| Endodontics | 0.870 | 1.391 | 0.039 | 0.626 | 0.532 | −1.875 to 3.615 | 0.637 | 1.570 |
| Oral and Maxillofacial Surgery | −1.377 | 1.549 | −0.055 | −0.889 | 0.375 | −4.434 to 1.680 | 0.660 | 1.516 |
| Oral and Maxillofacial Surgery Ward | 1.205 | 1.840 | 0.037 | 0.655 | 0.513 | −2.426 to 4.836 | 0.784 | 1.276 |
| Central Sterile Supply Department | 2.882 | 1.980 | 0.080 | 1.456 | 0.147 | −1.025 to 6.790 | 0.831 | 1.203 |
| Nursing Department | −4.437 | 3.196 | −0.073 | −1.388 | 0.167 | −10.745 to 1.871 | 0.915 | 1.093 |

***Note:*** The two observations were excluded because their absolute standardized residuals exceeded 3.0 in the primary model. The raw total Thriving at Work Scale score was the dependent variable, and all predictors were entered simultaneously. General Dentistry was the reference category for department of work. *B*, unstandardized regression coefficient; SE, standard error; *β*, standardized regression coefficient; *CI*, confidence interval. Department coefficients represent adjusted differences relative to General Dentistry. After the model was refitted, the maximum absolute standardized residual was 3.045; no further iterative case deletion was performed.
