# Supplementary material for: Thriving at Work and Associated Factors Among Nurses at a Chinese Stomatological Hospital: A Cross‐Sectional Study
Source: J Nurs Manag. 2026 Sep 15;2026:7301771. doi: 10.1155/jonm/7301771 (PMC13578935; doi:10.1155/jonm/7301771)
Supplement: Supplementary file 2 [file JONM-2026-7301771-s002.docx]

**Supplementary Table 2.** Sensitivity analysis of the multiple linear regression model after excluding the Nursing Department

**Model fit and diagnostics:** *N* = 186; R = 0.712; R2 = 0.506; adjusted R2 = 0.469; standard error of the estimate = 6.380; *F* (13, 172) = 13.572, *P* < 0.001; VIF range = 1.199-2.373; maximum Cook's distance = 0.104.

| **Variables** | ***B*** | **SE** | **Beta** | ***t*** | ***P*** | **95% *CI* for B** | **Tolerance** | **VIF** |
| --- | --- | --- | --- | --- | --- | --- | --- | --- |
| Constant | 28.539 | 4.798 |  | 5.948 | <0.001 | 19.069 to 38.008 |  |  |
| Neuroticism | -0.300 | 0.074 | -0.246 | -4.042 | <0.001 | -0.447 to -0.154 | 0.774 | 1.292 |
| Conscientiousness | 0.350 | 0.115 | 0.252 | 3.052 | 0.003 | 0.124 to 0.577 | 0.421 | 2.373 |
| Agreeableness | 0.230 | 0.114 | 0.158 | 2.020 | 0.045 | 0.005 to 0.454 | 0.471 | 2.121 |
| Openness | 0.000 | 0.085 | 0.000 | -0.002 | 0.998 | -0.167 to 0.167 | 0.593 | 1.687 |
| Extraversion | 0.184 | 0.082 | 0.143 | 2.251 | 0.026 | 0.023 to 0.345 | 0.707 | 1.414 |
| Empowering leadership total score | 0.112 | 0.071 | 0.102 | 1.578 | 0.116 | -0.028 to 0.253 | 0.692 | 1.444 |
| Pediatric Dentistry | -5.153 | 1.870 | -0.170 | -2.756 | 0.006 | -8.843 to -1.463 | 0.754 | 1.326 |
| Prosthodontics | 0.295 | 1.875 | 0.010 | 0.157 | 0.875 | -3.406 to 3.995 | 0.750 | 1.334 |
| Orthodontics | -2.296 | 1.966 | -0.072 | -1.168 | 0.244 | -6.176 to 1.584 | 0.764 | 1.309 |
| Endodontics | 0.743 | 1.441 | 0.034 | 0.516 | 0.607 | -2.101 to 3.586 | 0.649 | 1.542 |
| Oral and Maxillofacial Surgery | -1.865 | 1.625 | -0.075 | -1.148 | 0.253 | -5.073 to 1.342 | 0.668 | 1.498 |
| Oral and Maxillofacial Surgery Ward | 0.539 | 1.930 | 0.017 | 0.279 | 0.780 | -3.270 to 4.349 | 0.792 | 1.262 |
| Central Sterile Supply Department | 2.682 | 2.085 | 0.075 | 1.287 | 0.200 | -1.433 to 6.798 | 0.834 | 1.199 |

***Note:*** The Nursing Department was excluded because of its very small sample size (*n* = 4). The dependent variable was the raw total score of the Thriving at Work Scale. General Dentistry was used as the reference category for department of work. B, unstandardized regression coefficient; SE, standard error; Beta, standardized regression coefficient; *CI*, confidence interval; VIF, variance inflation factor. For department variables, coefficients represent adjusted differences compared with General Dentistry.
