# Supplementary material for: Thriving at Work and Associated Factors Among Nurses at a Chinese Stomatological Hospital: A Cross‐Sectional Study
Source: J Nurs Manag. 2026 Sep 15;2026:7301771. doi: 10.1155/jonm/7301771 (PMC13578935; doi:10.1155/jonm/7301771)
Supplement: Supplementary file 3 [file JONM-2026-7301771-s003.docx]

**Supplementary Table 3.** Sensitivity analysis additionally adjusting for years of service and daily working hours

| **Variables** | ***B*** | **SE** | ***β*** | ***P*** | **95% *CI* for B** | **VIF** |
| --- | --- | --- | --- | --- | --- | --- |
| Constant | 28.698 | 4.857 | — | <0.001 | 19.111 to 38.285 | — |
| Neuroticism | −0.321 | 0.074 | −0.262 | **<0.001** | −0.468 to −0.175 | 1.315 |
| Conscientiousness | 0.364 | 0.117 | 0.257 | **0.002** | 0.133 to 0.595 | 2.434 |
| Agreeableness | 0.184 | 0.113 | 0.125 | 0.106 | −0.039 to 0.407 | 2.128 |
| Openness | −0.014 | 0.086 | −0.011 | 0.873 | −0.184 to 0.157 | 1.724 |
| Extraversion | 0.205 | 0.083 | 0.157 | **0.015** | 0.040 to 0.370 | 1.459 |
| Empowering leadership total score | 0.144 | 0.069 | 0.133 | **0.038** | 0.008 to 0.280 | 1.447 |
| **Department of work (reference: General Dentistry)** |  |  |  |  |  |  |
| Pediatric Dentistry | −5.048 | 1.913 | −0.163 | **0.009** | −8.823 to −1.272 | 1.358 |
| Prosthodontics | 0.391 | 1.909 | 0.013 | 0.838 | −3.376 to 4.159 | 1.352 |
| Orthodontics | −2.800 | 2.257 | −0.085 | 0.216 | −7.255 to 1.655 | 1.687 |
| Endodontics | 0.492 | 1.487 | 0.022 | 0.741 | −2.444 to 3.428 | 1.612 |
| Oral and Maxillofacial Surgery | −1.656 | 1.650 | −0.065 | 0.317 | −4.913 to 1.602 | 1.513 |
| Oral and Maxillofacial Surgery Ward | 0.350 | 2.016 | 0.011 | 0.862 | −3.630 to 4.330 | 1.347 |
| Central Sterile Supply Department | 2.725 | 2.122 | 0.075 | 0.201 | −1.464 to 6.915 | 1.214 |
| Nursing Department | −4.949 | 3.421 | −0.080 | 0.150 | −11.703 to 1.805 | 1.099 |
| **Years of service (reference: 1–5 years)** |  |  |  |  |  |  |
| 6–10 years | −0.553 | 1.189 | −0.029 | 0.643 | −2.901 to 1.795 | 1.428 |
| ≥11 years | −0.500 | 1.231 | −0.026 | 0.686 | −2.930 to 1.931 | 1.436 |
| **Daily working hours (reference: 7–8 h)** |  |  |  |  |  |  |
| <7 h | 1.718 | 2.450 | 0.039 | 0.484 | −3.118 to 6.553 | 1.103 |
| >8 h | 0.952 | 1.459 | 0.043 | 0.515 | −1.927 to 3.832 | 1.551 |

***Note:*** *N* = 190. The raw total Thriving at Work Scale (TWS) score was the dependent variable. The primary model was additionally adjusted for years of service and daily working hours. Department, years of service, and daily working hours were represented by dummy variables, with General Dentistry, 1–5 years, and 7–8 h as the respective reference categories. All predictors were entered simultaneously. Model summary: R² = 0.522; adjusted R² = 0.472; *F* (18, 171) = 10.388; *P* < 0.001. Compared with the primary model, the additional covariates produced ΔR² = 0.003, *F* change (4, 171) = 0.267, *P* = 0.899. VIFs ranged from 1.099 to 2.434. The maximum Cook’s distance was 0.121, and standardized residuals ranged from −2.194 to 2.954. *B*, unstandardized regression coefficient; SE, standard error; *β*, standardized regression coefficient; *CI*, confidence interval; VIF, variance inflation factor.
